# Synergism in the effect of prior jasmonic acid application on herbivore-induced volatile emission by Lima bean plants: transcription of a monoterpene synthase gene and volatile emission

**DOI:** 10.1093/jxb/eru242

**Published:** 2014-06-13

**Authors:** Tila R. Menzel, Berhane T. Weldegergis, Anja David, Wilhelm Boland, Rieta Gols, Joop J. A. van Loon, Marcel Dicke

**Affiliations:** ^1^Laboratory of Entomology, Wageningen University, P.O. Box 8031, 6700 EH Wageningen, The Netherlands; ^2^Max Planck Institute for Chemical Ecology, Hans Knoell Strasse 8, D-07745 Jena, Germany

**Keywords:** Herbivore-induced plant volatiles, induced plant defence, mite–plant interactions, phytohormones, plant memory, terpene synthase, priming.

## Abstract

Jasmonic acid (JA) plays a central role in induced plant defence e.g. by regulating the biosynthesis of herbivore-induced plant volatiles that mediate the attraction of natural enemies of herbivores. Moreover, exogenous application of JA can be used to elicit plant defence responses similar to those induced by biting-chewing herbivores and mites that pierce cells and consume their contents. In the present study, we used Lima bean (*Phaseolus lunatus*) plants to explore how application of a low dose of JA followed by minor herbivory by spider mites (*Tetranychus urticae*) affects transcript levels of *P. lunatus* (*E*)-β-ocimene synthase (*PlOS*), emission of (*E*)-β-ocimene and nine other plant volatiles commonly associated with herbivory. Furthermore, we investigated the plant’s phytohormonal response. Application of a low dose of JA increased *PlOS t*ranscript levels in a synergistic manner when followed by minor herbivory for both simultaneous and sequential infestation. Emission of (*E)-*β-ocimene was also increased, and only JA, but not SA, levels were affected by treatments. Projection to latent structures-discriminant analysis (PLS-DA) of other volatiles showed overlap between treatments. Thus, a low-dose JA application results in a synergistic effect on gene transcription and an increased emission of a volatile compound involved in indirect defence after herbivore infestation.

## Summary

Low-dose JA application synergizes with effect of spider-mite induction of gene coding for ocimene synthase, and increased emission of (E)-β-ocimene, a compound known to attract carnivorous enemies of herbivorous spider-mites.

## Introduction

Plants possess a whole arsenal of mechanisms to resist attacks by pathogens and herbivorous arthropods. The basis of induced plant resistance against insect herbivory consists of a complex network of phytohormonal signalling. A general component of the response to chewing herbivores and foliar wounding is elicitation of the jasmonic acid (JA) signalling pathway in which the phytohormone JA plays a central role (McConn *et al.*, 1997; Kessler and Baldwin, 2002). In contrast, piercing-sucking insects and biotrophic pathogens commonly induce the salicylic acid (SA) signalling pathway, which antagonizes the JA pathway (Kempema *et al.*, 2007; Thaler *et al.*, 2012). Both pathways regulate large-scale changes in defence-related parts of the plant transcriptome, proteome, and metabolome, which underlie plant direct and indirect resistance mechanisms (Kessler and Baldwin, 2002; Pieterse and Dicke, 2007).

Biosynthesis of JA is initiated by the perception of herbivore- and damage-associated molecular patterns (HAMPs and DAMPs, respectively), which accompany herbivore attack and mechanical damage of plant tissue (Mithöfer & Boland 2008). The synthesis and accumulation of the JA–isoleucine conjugate, JA–Ile, generally causes a derepression of relevant transcription factors and defence-related genes in the plant (Boter *et al.*, 2004; Lorenzo *et al.*, 2004; Chini *et al.*, 2007; Thines *et al.*, 2007). Activation of these JA-responsive genes then leads to the production of metabolites involved in plant resistance. Local activation of JA signalling also results in the production of signalling molecules that can spread systemically through the plant and induce JA responses in distant organs, where they provide protection against imminent attackers (Ryan, 2000; Koo *et al.*, 2009). Although many processes within the JA pathway have been widely studied, the identity of specific gene products and metabolites that account for JA-mediated resistance are still unknown in most non-model plant species for which genomic sequence information is not yet available.

The role of the JA pathway in the regulation of induced plant volatile synthesis has been well studied. Early and late intermediates of the JA pathway as well as the final product, JA, induce synthesis of volatiles, which serve an important function in plant interactions with arthropods (Dicke *et al.*, 1999; Koch *et al.*, 1999; Bruinsma *et al.*, 2009a; Snoeren *et al.*, 2009; Bruinsma *et al.*, 2010). Volatile compounds that are synthesized *de novo* or in increased amounts by attacked plants are called herbivore-induced plant volatiles (HIPVs). These compounds are particularly involved in mediating tritrophic interactions, in which natural enemies of herbivores use plant volatiles as cues to locate their herbivorous host or prey (Mumm and Dicke, 2010). Although many of these compounds have been identified, another level of complexity is posed by the fact that the exact expression of the defence response by a plant is often modulated by the ecological context. Timing, intensity, and other characteristics of the defence response are influenced by factors such as the specific nature of the attacker (Takabayashi *et al.*, 1995; De Moraes *et al.*, 1998; Stout *et al.*, 1998; De Vos *et al.*, 2005), ontogenetic stage of the attacked plant (Hare, 2010) and plant tissue (Wentzell and Kliebenstein, 2008), and population density of plants and density of attackers (Gols *et al.*, 2003; Wentzell and Kliebenstein, 2008; Kegge *et al.*, 2013). Moreover, plant defences are further modulated by the simultaneous presence of multiple herbivores and pathogens on the same plant (Moayeri *et al.*, 2007; Dicke *et al.*, 2009), as well as previous infestations (Stout *et al.*, 1998; Jung *et al.*, 2009; Ponzio *et al.*, 2013).

Exogenous application of key phytohormones in defence signalling pathways can be used to elicit plant defence responses similar to those induced by arthropod herbivores or pathogens (Dicke *et al.*, 1999; Gols *et al.*, 1999; Koornneef *et al.*, 2008). Treatment of plants with JA, or its volatile derivative methyl jasmonate (MeJA), has been shown to confer broad resistance against plant attackers such as nematodes (Cooper *et al.*, 2005), biting-chewing insects (Omer *et al.*, 2000; Tierranegra-García *et al.*, 2011), and necrotrophic pathogens (Brader *et al.*, 2001; Yamada *et al.*, 2012). Even plants grown from seeds previously exposed to JA, have been found to be more resistant to herbivory (Worrall *et al.*, 2012). Observed JA-mediated resistance is attributed to enhanced induction of direct resistance mechanisms, such as accumulation of plant toxins or proteinase inhibitors, or indirect resistance mechanisms, that promote the effectiveness of natural enemies of plant attackers. Generally, application of JA induces volatile blends that are similar to those induced by herbivory (Dicke *et al.*, 1999; Gols *et al.*, 1999; Kessler and Baldwin, 2001). These volatile blends consist of compounds that can be exploited by natural enemies as cues to locate their herbivorous prey or host. Several studies have investigated the effect of phytohormonal induction on indirect resistance (e.g. Dicke and Vet, 1999; Gols *et al.*, 1999; Ozawa *et al.*, 2000; Bruinsma *et al.*, 2008; Bruinsma *et al.*, 2009b). Phytohormone application allows for manipulation of defined steps in signal-transduction pathways and to induce plants in a dose-controlled manner without removal of plant tissue.

In the present study, we have explored how a low JA-dose affects Lima bean indirect defence against the generalist herbivorous mite *Tetranychus urticae*. JA is a key regulator of the induction of volatiles emitted in response to *T. urticae* infestation such as (*E*)-β-ocimene (Dicke *et al.*, 1999; Ament *et al.*, 2004). The monoterpene (*E*)-β-ocimene is an HIPV released in response to herbivory by a range of plant species including cucumber, apple, Lima bean, cotton, corn, and tobacco (Paré and Tumlinson, 1999). Moreover, (*E*)-β-ocimene is one of the five principle compounds that mediate the attraction of the specialist predator *Phytoseiulus persimilis* to *T. urticae-*infested plants (Dicke *et al.*, 1990; De Boer and Dicke, 2004).

Gols *et al.* (2003) found that treatment of Lima bean plants with a low dose of JA, which in itself did not result in attraction of the predatory mite *P. persimilis,* resulted in an enhanced attraction of *P. persimilis* in response to herbivory by a low density of spider mites. Enhanced predator attraction was still found when a time lapse of 7 days was introduced between the treatment with JA and the infestation of spider mites. Here, we investigated the underlying mechanism. We hypothesized that exogenous application of a low dose of JA to Lima bean would induce JA-responsive gene transcription and subsequent terpene emissions with a priming or additive effect when followed by minor herbivory. We have focused on the transcription of the *Phaseolus lunatus* occimene synthase (*PlOS*) gene. *PlOS* codes for the enzyme ocimene synthase that mediates the rate-limiting step in the biosynthesis of (*E*)-β-ocimene (Ament *et al.*, 2004; Arimura *et al.*, 2004).

## Materials and methods

### Plants and mites

Lima bean plants (*Phaseolus lunatus* L., cv Wonderbush) were sown and grown in a greenhouse compartment at 23±2 °C with 60±10% R.H., and a photoperiod of 16L:8D. Plants having two fully expanded primary leaves were used for experiments at 12–15 d after sowing. Two-spotted spider mites, *Tetranychus urticae* Koch (Acari: Tetranychidae), were reared on Lima bean plants in a different greenhouse compartment under the same conditions as the Lima bean plants. Only adult female mites were used for experiments.

### Treatments

Primary leaves of Lima bean plants were sprayed with 1ml per leaf of 0.1mM JA solution (Sigma-Aldrich) in water or with 1ml of water as a control. The plants were left to dry for 30–60min. After phytohormone or control treatment, plants were transferred to a climate chamber and incubated separated by treatment in cages (metal frame 90×90×60cm, walls of polyethylene sheet) at 23±2 °C, 60±10% RH and 16L:8D. Each cage contained 16 plants per treatment for gene transcription and phytohormone analysis or four plants per treatment for volatile trapping experiments. The building’s vacuum system was connected to the top of each cage with a suction of approximately 7 l min^–1^ to avoid interactions through volatiles between plants of different treatments.

The four treatments were: (i) water, (ii) water and mites, (iii) JA, and (iv) JA and mites. For simultaneous infestations, spider mites were applied after plants sprayed with JA solutions were dry. Four adult female mites were evenly distributed over the two primary leaves of plants from the respective treatments using a fine paint brush. Mites were randomly selected from the spider-mite culture. After 2 d of incubation, the mites and their products (webbing, eggs) were removed using a fine paint brush.

In subsequent experiments with sequential infestation, mites were inoculated 7 d after JA treatment and transferred to cages as described above. 2 days before mite application, lanolin paste was applied around the petioles of both primary leaves of each plant to confine the mites to the leaves. After a seven day incubation period, leaf material from plants of treatments (i) water and (iii) JA was collected. The two other treatments, (ii) water and mites and (iv) JA and mites, received the mite treatment (four adult females per plant) and were incubated for another 2 d, after which leaf material was collected.

### RNA extraction and cDNA synthesis

Leaf material was collected by excising four leaf discs at 12.00–13.00h from a primary leaf using a cork borer (diameter 2cm), and the leaf discs obtained from three plants were pooled to give one biological replicate. Upon collection, samples were immediately shock-frozen in liquid nitrogen and stored at –80 °C until processing. The leaf material was homogenized without thawing using a mortar and pestle. Total RNA was extracted and purified using the Qiagen RNeasy Plant Mini kit with integrated DNAse treatment, following the manufacturer’s instructions. Absence of genomic DNA contamination and RNA quality were assessed using Agilent 2100 Bioanalyzer with the RNA 6000 Nano Labchip® kit (all from Agilent Technologies). RNA was quantified with a NanoDrop® ND-1000 spectrophotometer (NanoDrop Technologies, Wilmington, DE, USA). Only RNA samples with 260/280 wavelength ratio >2 and a RIN value >7 were used for cDNA synthesis. cDNA was generated from total RNA by using the Bio-Rad iScript cDNA synthesis kit (Bio-Rad), following the manufacturer’s instructions.

### Quantitative RT-PCR

Transcript levels of *P. lunatus Ocimene Synthase* (*PlOS;* GenBank accession EU194553) and the two reference genes *P. lunatus Actin1* (*PlACT1;* GenBank accession DQ159907) and *P. lunatus Nuclear matrix protein 1* (*PlNMP1*; GenBank accession AF289260.1) were quantified by performing a real-time quantitative RT-PCR in a Rotor-Gene 6000 machine (Corbett Research) with a 72-well rotor. Reactions were performed in a final volume of 25 µl, that included 12 µl iQ^TM^ SYBR^®^ Green Supermix (Bio-Rad), 1 µl forward primer (4 µM) and reverse primer (4 µM) pairs (final primer concentration: 160nM), and 5 µl cDNA (4ng µl^–1^) first strand template. The PCR program for *PlOS* and the reference gene *PlACT1* was the same as described by Zheng *et al.* (2007). The *PlOS* primers were F-*PlOS*5′- TGCATGGGTCTCAGTCTCTG-3′ and R-*PlOS*5′- TGCTGCTTCCCCTCTCTCTA-3′ with a predicted product length of 189bp. *PlACT1* primers were F-*PlACT1* 5′-CCAAGGCTAACCGTGAAAAG-3′ and R-*PlACT1*5′-AGC CAGATCAAGACGAAGGA-3′ with predicted product length of 208bp. The second reference gene, *PlNMP1*, was designed with the Geneious software version 4.8.3 under default parameters except that the annealing temperature was set to 56 °C. Predicted product length of the *PlNMP1* primers F-*PlNMP1* 5′-CCGGAATGGAGTGTTGACGAGCA-3′ and *R-PlNMP1* 5′-CCAGCT CAGAAACATCTGGCAATGG-3′ was 157bp. The PCR program for *PlNMP1* was adapted from Zheng *et al.* (2007), whereby the extension time was increased from 45–48 s. Specificity of amplicons was verified for each primer pair by melt-curve analysis to assure absence of non-specific products as well as primer-dimer formation. Relative quantification of *PlOS* transcription was calculated with the 2^–∆∆Ct^ method (Livak and Schmittgen, 2001), using a normalization factor (Vandesompele *et al.*, 2002). The normalization factor was calculated by geometrically averaging the threshold cycle (Ct) values from the two reference genes *ACT1* and *NMP1* (M<0.03, GeNorm). Subtraction of the normalization factor from *PlOS* Ct values normalizes for differences in cDNA synthesis.

### Phytohormone quantification

Quantification of JA and SA levels in samples used for gene transcription analysis followed the protocol of Schulze *et al.* (2006). Samples were analysed on a Finnigan ITQ Instrument (Thermo Electron, Bremen, Germany) running in a CI-negative ion mode.

### Dynamic headspace collection of plant volatiles

Collection of plant volatiles was carried out in 20-l glass jars sealed with a viton-lined glass lid with an inlet and outlet. Compressed air was filtered by passing through charcoal before entering the glass jar containing the plant. Volatiles were collected by sucking air out of the glass jar at a constant rate of 200ml min^–1^ through a stainless steel tube filled with 200mg Tenax TA (Markes, Llantrisant, UK) for 2h. Before sampling, empty glass jars were purged with compressed air for 1h. Pots in which the plants had grown were removed, roots and soil were carefully wrapped in aluminium foil, and then the plant was placed in a glass jar. The glass jars containing the plants were flushed for an additional 30 min before connecting stainless steel tubes filled with Tenax TA. Plant volatiles were collected from seven replicates of each of the treatments: (i) water, (ii) water and mites, (iii) JA, and (iv) JA and mites. Fresh weight of above-ground plant tissue was determined immediately after volatile collection using an analytical balance (NewClassic ML, Mettler Toledo, Switzerland).

### Analysis of plant volatiles

Thermo Trace GC Ultra coupled with Thermo Trace DSQ quadrupole mass spectrometer (Thermo Fisher Scientific, Waltham, USA) was used for separation and detection of plant volatiles. Before release of the volatiles, each sample was spiked with 10ng µl^–1^ of 1-bromodecane as internal standard (I.S.) and dry-purged under a stream of nitrogen (50ml min^–1^) for 10min at ambient temperature to remove moisture and the organic solvent methanol used to prepare the I.S. The collected volatiles and I.S. were released from the Tenax TA using the Ultra 50:50 thermodesorption unit (Markes) at 250 °C for 10min under helium flow of 20ml min^–1^, while re-collecting the volatiles in a thermally cooled universal solvent trap at 10 °C using Unity (Markes). Once the desorption process was completed, volatile compounds were released from the cold trap by ballistic heating at 40 °C s^–1^ to 280 °C. The temperature was kept at 280 °C for 10min, while the volatiles were transferred to a ZB-5MSi analytical column [30 m×0.25mm I.D.×1.00 µm F.T. (Phenomenex, Torrance, CA, USA)], in a splitless mode for further separation. The GC oven temperature was initially held at 40 °C for 2min and was raised at 10 °C min^–1^ to a final temperature of 280 °C, where it was kept for 4min under a helium flow of 1ml min^–1^ in a constant flow mode. The DSQ mass spectrometer (MS) was operated in a scan mode with a mass range of 35–350 amu at 5.38 scans s^–1^ and spectra were recorded in electron impact ionisation (EI) mode at 70eV. MS transfer line and ion source were set at 275 and 250 °C, respectively. Compound identification was based on retention time of authentic standards and comparison of mass spectra with those in the NIST 2005 and Wageningen Mass Spectral Database of Natural Products MS libraries. Experimentally calculated linear retention indices (LRI) were also used as additional measure to confirm the identity of compounds.

Standards of (*E*)-2-hexenal, (*Z*)-3-hexen-1-ol, (*Z*)-3-hexen-1-ol acetate, (*E*)-β-ocimene, linalool, methyl salicylate (MeSA), indole, caryophyllene as well as the internal standard (I.S.) 1-bromodecane, a series of alkane mixtures (C8–C20) and the solvent methanol (GC grade) were obtained from Sigma-Aldrich (Saint Louis, MO, USA). Additional standards (*E*)-4,8-dimethylnona-1,3,7-triene [(*E)-*DMNT] and (*E,E*)-4,8,12-trimethyltrideca-1,3,7,11-tetraene [(*E,E)-*TMTT] were synthesized at the Max Planck Institute of Chemical Ecology (Jena, Germany) following the procedure by Boland and Gäbler (1989). For quantification, calibration lines were constructed for each compound using seven data points at different concentrations (two replicates of each data point) and was carried out using a single (target) ion, in selected ion monitoring (SIM) mode.

### Statistical analysis

Univariate data, i.e. gene transcription and plant volatile data, were log-transformed to meet the test assumptions of normality and homogeneity of variances. Phytohormone data were analysed without transformation. Analyses were performed using one-way ANOVA followed by Fisher’s least significant difference (LSD) post-hoc tests for pair-wise comparisons between treatments in the statistical software SPSS version 19 (SPSS Inc., Chicago, IL, USA). If assumptions on normality and equal variance were violated, Kruskal-Wallis tests followed by Mann-Whitney U tests with a Bonferroni correction as post-hoc tests were used. Assumption of synergism was tested by subtraction of baseline levels of both single treatments and subsequent summation. If the resulting value was outside the 95% confidence interval of the mean from a combination treatment, the interaction between the single treatments was considered significantly different.

Effects of treatments, time of trapping, and the interaction on (*E*)-β-ocimene emission were analysed by general linear model (GLM) with LSD post-hoc tests. Evaluation of differences between treatments of morning trapping and afternoon trapping were done by a one-way ANOVA followed by Fisher’s least significant difference (LSD) post-hoc tests for pair-wise comparisons.

The multivariate data analysis of plant volatiles corrected by fresh weight using projection to latent structures-discriminant analysis (PLS-DA) was performed to test for differences in volatile profiles among different treatments. The analysis was carried out using the software SIMCA P+ version 12 (Umetrics, Umeå, Sweden). Data were log-transformed and univariate-scaled prior to PLS-DA analysis.

## Results

### Transcriptional changes in *PlOS* levels in response to JA and spider-mite treatment

**Fig. 1.**
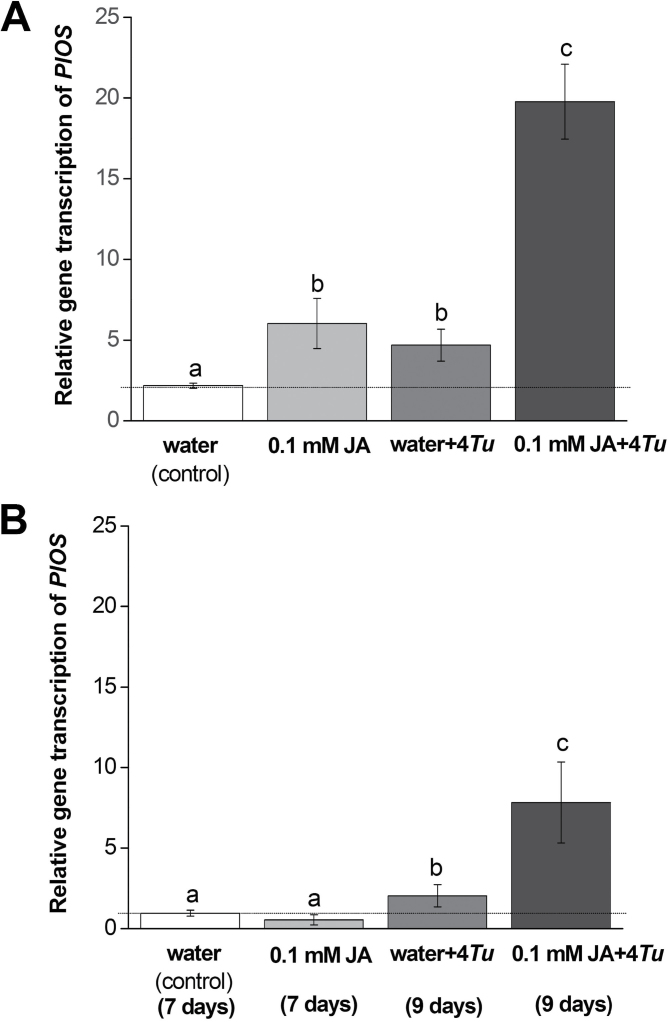
Relative gene transcript levels of *PlOS* quantified in *P. lunatus* plants treated with (i) water (control), (ii) 0.1mM JA, (iii) four *T. urticae* (water+4*Tu*), or (iv) 0.1mM JA with four *T. urticae* mites (0.1mM JA+4*Tu*). (A) Inoculation of four adult female *T. urticae* on plants was done immediately following JA-treatment and mites had been feeding for 48h, and (B) inoculation of four adult female *T. urticae* was done 7 days after incubation with water or 0.1mM JA and mites had been feeding for 48h. Values are the mean (± SE) of three to four biological replicates, different letters above bars indicate significant differences in transcript levels between treatments (ANOVA followed by Fisher’s LSD test, α=0.05). *PlOS* transcript levels were normalized to the normalization factor obtained from geometrically averaging the Ct values of the two reference genes *PlACT1 and PlNMP1* for each sample. Baseline represents transcript level in control plants.

Transcript levels of *PlOS* in response to the treatments, i.e. (i) water (control), (ii) 0.1mM JA, (iii) four *T. urticae,* and the combined treatment (iv) 0.1mM JA with simultaneous inoculation of four *T. urticae* showed significant differences (Fig. 1A).

Plants treated with 0.1mM JA or four *T. urticae* alone showed higher (*P*<0.05 for both comparisons) *PlOS* transcript levels after 48h compared with control plants, but did not differ from each other. Plants treated with the combination of 0.1mM JA and four simultaneously inoculated *T. urticae* also showed higher (*P*<0.01) *PlOS* levels after 48h compared with control and the single treatment with JA or mites. The combination treatment resulted in a *PlOS* transcript level that is twice the level that would be obtained if the effects of JA and four *T. urticae* were additive, revealing a synergistic effect of the two treatments on *PlOS* transcript levels.

Significant differences between treatments were also found in the second experiment in which inoculation of *T. urticae* was done 7 days after the application of 0.1mM JA or water (*P*<0.05; Fig. 1B). *PlOS* transcript levels in plants treated with 0.1mM JA were not significantly different from control plants after 7 days of incubation. When four *T. urticae* were inoculated on water-treated plants at this time point and incubated for another 2 days, the *PlOS* transcript level was significantly higher (*P*<0.05) compared with 0.1mM JA treatment alone. After 7 days of incubation, plants treated with the combination of 0.1mM JA and four *T. urticae* for 2 days showed higher *PlOS* levels compared with control, 0.1mM JA, and four *T. urticae* treatment alone (*P*<0.05 for all comparisons). Compared with 0.1mM JA or four *T. urticae* alone, the combination had a higher *PlOS* level than would be obtained from additive effects of four *T. urticae* and 0.1mM JA, indicating a synergistic effect of the two treatments on *PlOS* transcript levels.

This experiment has been repeated two and three more times respectively and the results were consistent with those presented in Fig. 1. See Supplementary Fig. 1 and 2 at JXB online for the results.

### Phytohormone levels

**Fig. 2.**
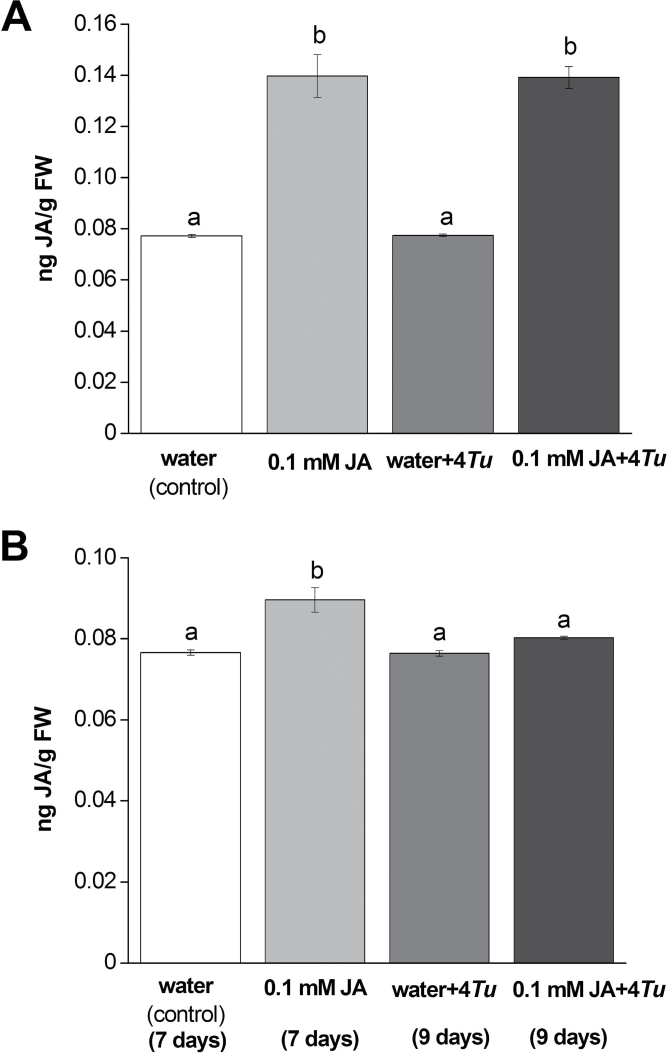
JA levels in ng JA per g FW in *P. lunatus* plants treated with (i) water (control), (ii) 0.1mM JA, (iii) four *T. urticae* (water+4*Tu*), or (iv) 0.1mM JA with four *T. urticae* mites (0.1mM JA+4*Tu*). (A) Plants were inoculated with four adult female *T. urticae* immediately after JA treatment and incubated for 48h, and (B) plants were inoculated with four adult female *T. urticae* 7 days after JA treatment and incubated for an additional 48h. Values are the mean (±SE) of four biological replicates, and were analysed by Kruskal-Wallis test (A) or ANOVA (B) respectively (α=0.05).

We investigated the effects of single treatments (i) water (control), (ii) 0.1mM JA, (iii) four *T. urticae,* and (iv) the combined treatment of 0.1mM JA with simultaneous inoculation of four *T. urticae* on JA levels (Fig. 2). A significant treatment effect was found (*P=*0.01; Fig 2A). Application of 0.1mM JA resulted in higher JA levels at 48h compared with control plants. Four *T. urticae*, however, did not increase JA levels in the plants compared with the control treatment. Plants treated with the combination of 0.1mM JA and simultaneously four *T. urticae* also showed higher JA levels compared with control, but not different from 0.1mM JA treatment alone.

Significant differences in JA levels were also found among treatments when mites had been inoculated 7 days after JA or water application (*P*<0.01; Fig 2B). After 7 days of incubation with 0.1mM JA there is still an increase (*P*<0.001) in JA level compared with control. The combination of 0.1mM JA application and inoculation of *T. urticae* 7 days later that had been feeding for 2 days resulted in JA levels after 9 days that were similar to that of the control treatment. The introduction of four *T. urticae* alone did not affect JA levels.

No treatment effect was found for SA levels between control and other treatments for simultaneous (*P=*0.81; Supplementary Fig. 3A at JXB online) or sequential mite application (*P=*0.33; Supplementary Fig. 3B at JXB online).

### Volatile emission

**Fig. 3.**
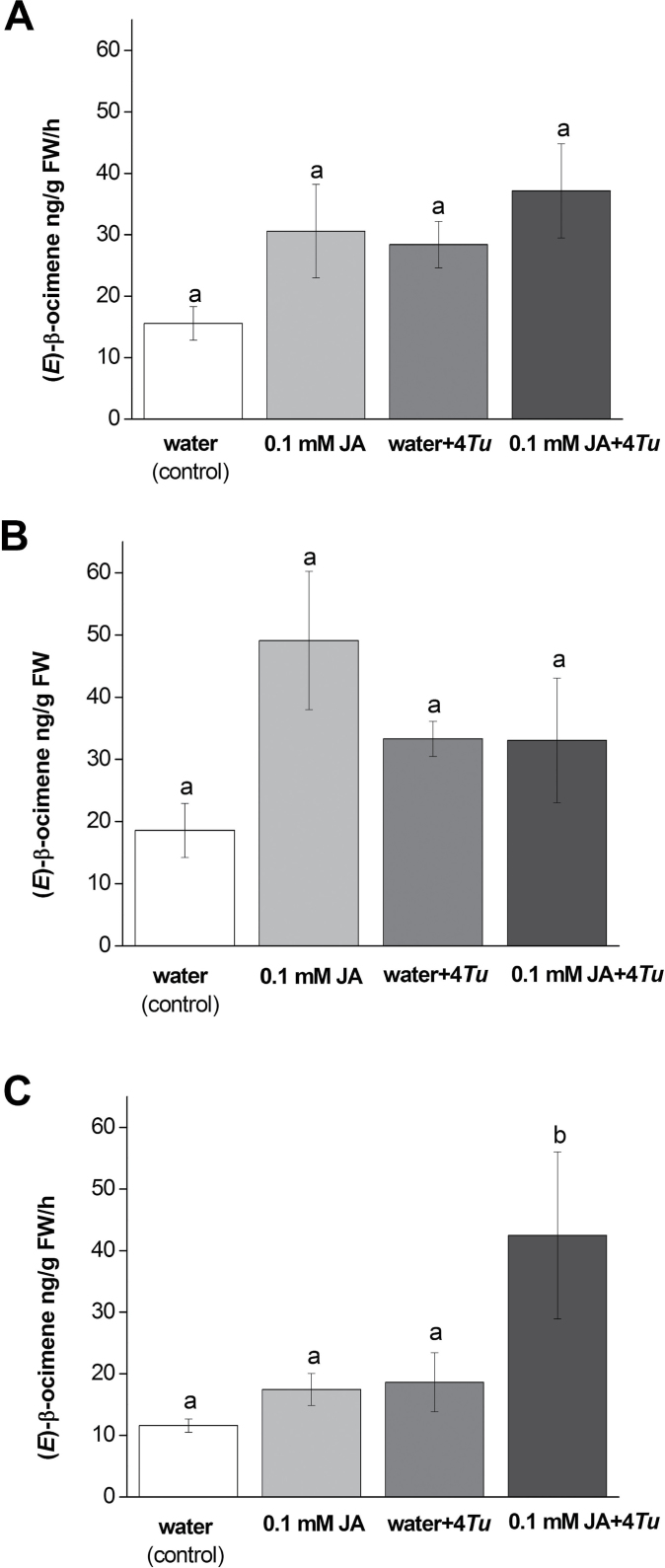
Average (*E*)-β-ocimene emission rates in ng per g FW h^–1^ after four different treatments of *P. lunatus* plants. Treatments were (i) control (water), (ii) 0.1mM JA, (iii) four *T. urticae* (water+4*Tu*), or (iv) 0.1mM JA with four *T. urticae* mites (0.1mM JA+4*Tu*) inoculated immediately after JA application and incubated for 48h. (A) depicts combined morning and afternoon trappings, (B) morning trappings only (ca. 11.00–13.00h), and (C) afternoon trappings only (ca. 14.00–16.00h). Values are the mean (± SE) of six to seven biological replicates for (A), and three to four biological replicates for (B) and (C), except for water+4*Tu* in (C) with two biological replicates. Different letters above bars indicate significant differences in emission rates between treatments (Fisher’s LSD tests, α=0.05).

Emission rates of the monoterpene (*E*)-β-ocimene were compared among treatments and time of trapping of the simultaneous *T. urticae* application experiment. There was a treatment effect (*P<*0.05), however, although emission rates of plants treated with 0.1mM JA, mites, or both, were higher than control treatment, the post-hoc test did not yield statistical differences among treatments (*P*>0.05; Fig. 3A). However, the time of trapping (morning, i.e. ca. 11.00–13.00h or afternoon, i.e. ca. 14.00–16.00h) may also have an effect. Volatile trappings executed during mornings showed no overall effect of treatments (*P=*0.20; Fig. 3B). In afternoon trappings, however, a treatment effect was found (*P=*0.02; Fig. 3C), and plants treated with 0.1mM JA and four *T. urticae* showed increased (*E*)-β-ocimene emission compared with other treatments (*P<*0.05).

**Fig. 4.**
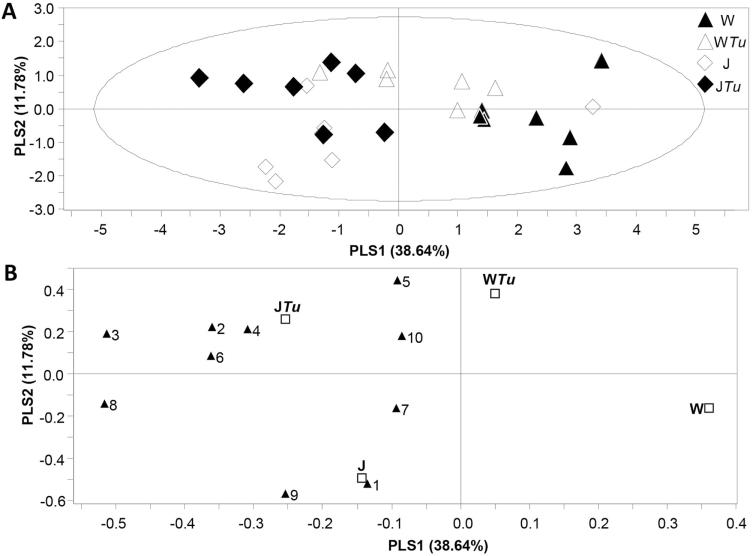
Multivariate data analysis by PLS-DA and corresponding loading plot of targeted volatiles of *P. lunatus* plants exposed to (i) water (control, W), (ii) 0.1mM JA (J), (iii) water and four *T. urticae* spider mites (W*Tu*), or combined treatment (iv) 0.1mM JA with immediate application of four *T. urticae* (J*Tu*). (A) PLS-DA score plot showing the ordination of the samples according to the first two PLS components based on the quantitative values of volatiles between different treatments. Explained variance by first and second PLS components is given in brackets. Loading plot (B) shows the contribution of each volatile to the discrimination between treatments using the first two PLS components. Numbers represent: 1, (*E*)-2-hexenal; 2, (*Z*)-3-hexen-1-ol; 3, (*Z*)-3-hexen-1-ol acetate; 4, (*E*)-β-ocimene; 5, linalool; 6 (*E*)-4,8-dimethyl-1,3,7-nonatriene [(*E*)-DMNT]; 7, methyl salicylate (MeSA); 8, indole; 9, β-caryophyllene; 10, (*E,E*)-4,8,12-trimethyltrideca-1,3,7,11-tetraene [(*E,E*)-TMTT]. Squares represent the four treatments (labelled *W, WTu, J,* and *JTu*).

Emission of a total of the ten major volatile compounds was also compared among the treatments (Fig. 4). These ten compounds were (*E*)-2-hexenal, (*Z*)-3-hexen-1-ol, (*Z*)-3-hexen-1-ol acetate, (*E*)-β-ocimene, linalool, methyl salicylate, indole, β-caryophyllene, (*E*)-DMNT, and (*E,E*)-TMTT. They constitute well-known herbivore-induced plant volatiles (HIPV) observed in *T. urticae-*infested Lima bean plants (Dicke *et al.*, 1990; Dicke *et al.*, 1999). PLS-DA including all four treatments resulted in a model with one significant component, whereby volatile blends emitted by control (water-treated) plants clearly differed from those emitted by plants exposed to the other three treatments. The volatile emission profiles of plants exposed to the combined 0.1mM JA plus four *T. urticae* treatment overlapped to a large extent with those of plants exposed to 0.1mM JA alone. Volatile blends emitted by plants exposed to four *T. urticae* exhibited similarities with those from control plants, but also with those from 0.1 mM JA-treated plants. Treatment of plants with JA, mites, or a JA-mite combination increased the emission of all ten volatiles (Fig. 4B). Compared with the control treatment, treatment of plants with JA (J and J*Tu,* Fig. 4B) resulted in higher emissions of indole, the green leaf volatiles (*Z*)-3-hexen-1-ol acetate and (*Z*)-3-hexen-1-ol, and to a lesser extent the terpenoids (*E*)-DMNT, (*E*)-β-ocimene, as well as β-caryophyllene. The emission rates of the latter three compounds were intermediate in plants exposed to mites alone.

**Fig. 5.**
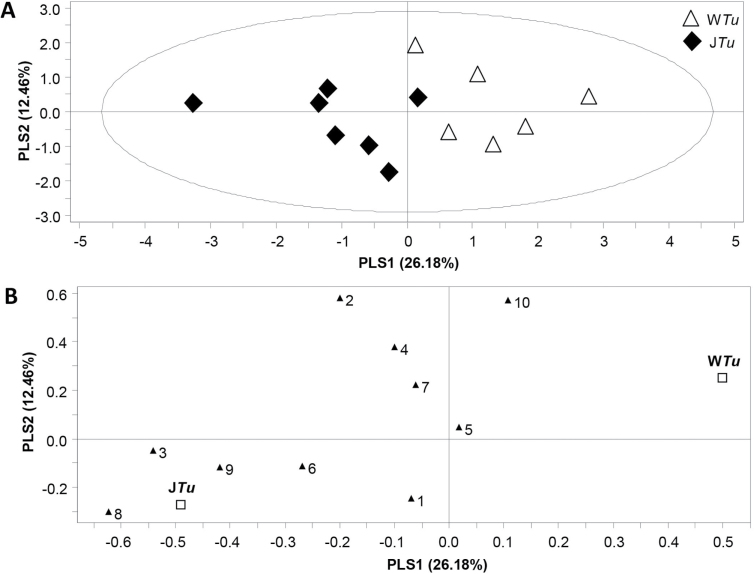
Multivariate data analysis using PLS-DA and corresponding loading plot of volatile compounds emitted by *P. lunatus* plants subjected to either four *T. urticae* (W*Tu*) or the combination of 0.1mM JA and four *T. urticae* (J*Tu*). The score plot (A) visualises the separation pattern of the samples according to their classes using the first and second PLS component with the explained variance in brackets and the loading plot (B) depicts the contribution of volatiles to the class separation using the first two PLS components. The second PLS component was not significant and is only shown for representational purposes. Numbers represent: 1, (*E*)-2-hexenal; 2, (*Z*)-3-hexen-1-ol; 3, (*Z*)-3-hexen-1-ol acetate; 4, (*E*)-β-ocimene; 5, linalool; 6 (*E*)-4,8-dimethyl-1,3,7-nonatriene [(*E*)-DMNT]; 7, methyl salicylate (MeSA); 8, indole; 9, β-caryophyllene; 10, (*E,E*)-4,8,12-trimethyltrideca-1,3,7,11-tetraene [(E,E)-TMTT]. Squares represent the two treatments (labelled *WTu* and *JTu*).

A pairwise comparison of volatile profiles from treatments including mites, i.e. water plus four *T. urticae* (W*Tu*) and combined 0.1mM JA treatment plus four *T. urticae* (J*Tu*) resulted in a significant PLS-DA model with one significant component (Fig. 5). Pre-treatment with JA before *T. urticae* infestation resulted in a plant volatile profile that was separate from the profile of plants without the JA treatment.

## Discussion

In their natural environment plants are frequently exposed to multiple herbivory, whereby herbivores may arrive simultaneously or separated in time. Both types of infestations may influence the plant phenotype and therefore affect tritrophic interactions with natural enemies involved in plant indirect defence. Here, we used the phytohormone JA followed by herbivory by a low number of herbivores to study the effects of this phytohormone on transcript levels of (*E*)-β-ocimene synthase, emission of the corresponding volatile compound, and other volatiles commonly emitted from plants in response to simultaneous and sequential herbivory. The volatile organic compound (*E*)-β-ocimene plays an important role in plant indirect defence in many plant species, including Lima bean, by attracting natural enemies of herbivorous arthropods (Dicke *et al.*, 1990; Arimura *et al.*, 2000; Arimura *et al.*, 2002; Zhang *et al.*, 2009a; Muroi *et al.*, 2011).

We found that Lima bean plants treated with a low dose of JA exhibited increased *PlOS t*ranscript levels in a synergistic manner when followed by minor herbivory, irrespective of the herbivory occurring simultaneously or sequentially. Accordingly, Gols *et al.* (2003) found that plants treated with a low dose of JA followed by simultaneous or sequential minor herbivory by *T. urticae* were highly attractive to the predatory mite *P. persimilis*: the predators preferred volatiles emitted from plants treated with 0.1mM JA and infested with four *T. urticae* over volatiles from plants infested with only four *T. urticae*. Quantification of (*E)-*β-ocimene emission in the headspace of Lima bean plants shows that the emission rate of the volatile itself was also increased in combination treatments. The increase was only significant during the afternoon. The latter connects to findings of Arimura *et al.* (2008) that show that (*E*)-β-ocimene emission rates increase from the onset of light and peak during the afternoon after herbivory or leaf damage. Generally, (*E*)-β-ocimene seems to play an important role in the attraction of *P. persimilis* in plant interactions with multiple herbivores. For instance, De Boer *et al.* (2008) found that (*E*)-β-ocimene emission and predator attraction were increased in a synergistic manner in response to simultaneous infestation by prey and non-prey herbivores on a Lima bean plant. Moreover, Zhang *et al.* (2009b) showed that feeding by a non-prey herbivore, i.e. whiteflies, negatively affected (*E*)-β-ocimene emission and corresponding transcript levels of *PlOS*, which resulted in decreased attraction of *P. persimilis* to Lima bean plants simultaneously infested with spider-mites and whiteflies. The main underlying mechanism seems to be phytohormone induction and crosstalk among them. Whiteflies induce SA, which antagonizes the JA pathway, whereas caterpillars and spider mites mainly induce the JA pathway (Blechert *et al.*, 1995; Arimura *et al.*, 2002; Schmelz *et al.*, 2003). In our study we found a synergistic effect of a low dose 0.1mM JA and a low density infestation by four *T. urticae* on *PlOS* transcript levels after 48h of spider-mite infestation. In the case of a 7-day delay between JA treatment and spider-mite inoculation, JA did not induce *PlOS* transcription, but in combination with spider mite feeding resulted in an enhanced transcription compared with spider-mite induction alone. Thus, in this case JA had primed the transcription of this gene. Yet JA levels were similar for JA-treated plants and plants induced with both JA and *T. urticae.* Even when JA titres and *PlOS* transcript levels returned to control levels, subsequent mite infestation still increased *PlOS* transcript levels to higher values than recorded after mite infestation alone. Introduction of a time lag between first induction of plant defence by JA and a second induction by herbivory did not impair plant ability for enhanced defence induction. In fact, this corresponds with behavioural results reported by Gols *et al.* (2003) for the predatory mite *P. persimilis*, which was more strongly attracted to sequentially induced plants than to plants only induced by spider mites. It has been previously suggested that plants are able to form some sort of memory, sometimes called a “primed state”, which enables them to accelerate and/or enhance defence responses to a second challenge (Frost *et al.*, 2008; Conrath, 2009). Maintenance of plant defence is thought to entail costs and is ineffective in the absence of herbivores. Consequently, plants have developed defence mechanisms that are inducible by herbivory (Heil and Baldwin, 2002). In the case of priming, costly defence metabolites are not produced immediately upon a minor challenge, thereby considerably reducing the cost of this mechanism (Van Hulten *et al.*, 2006; Walters *et al.*, 2008; Perazzolli *et al.*, 2011). In our experiments, previous induction of *PlOS* by JA seemed to sensitize the gene in such a way that a second challenge using a small number of herbivores at a later time point resulted in increased transcript levels. The ability of phytohormones to generate a primed state in terms of enhanced defence gene transcription has previously been reported for e.g. SA and the SA-analogue benzothiadiazole (BTH) in *Petroselinum crispum* L. and *Arabidopsis thaliana* (Thulke and Conrath, 1998; Kohler *et al.*, 2002).

Natural enemies of herbivores respond to mixtures of HIPV rather than to a single volatile. Blends can carry information on e.g. herbivore identity or herbivore developmental stage (Takabayashi *et al.*, 1995; De Moraes *et al.*, 1998; Stout *et al.*, 1998; De Vos *et al.*, 2005; Mumm and Dicke, 2010). JA application is known to induce a volatile blend that is similar to the blend induced by *T. urticae* mites (Dicke *et al.*, 1999; Gols *et al.*, 1999). However, defence induction by JA seems to be more generic and natural enemies often prefer HIPVs induced by actual hosts or prey over JA-induced plants (Van Poecke and Dicke, 2002; De Boer and Dicke, 2004; Ozawa *et al.*, 2004; Bruinsma *et al.*, 2008; Bruinsma *et al.*, 2009b). Our targeted chemical analysis comparing the volatile profiles of 10 well-known major HIPVs emitted by Lima bean plants among treatments showed indeed a large overlap for JA- and mite-treated plants and a clear separation from the blend emitted by control plants. However, Gols *et al.* (2003) found that volatiles emitted by Lima bean plants in response to a low dose of 0.1mM JA do not attract the predator *P. persimilis,* whereas a low infestation density of four *T. urticae,* and particularly the combination of treatments, does. Qualitative and quantitative differences in volatile blends must thus affect the behaviour of the predatory mite. Volatile emission profiles of plants with herbivores with and without simultaneous JA treatment do not only show a great overlap, but also demonstrated that other volatiles, besides (*E*)-β-ocimene, are likely to determine attractiveness of the volatile blend attractive to *P. persimilis*. Although (*E*)-β-ocimene is known to be an important host location cues in Lima bean, De Boer *et al.* (2004) found that (*E*)-β-ocimene is also emitted in response to caterpillar feeding. Predators must therefore gain additional information from other HIPVs, such as MeSA and (*E,E)-*TMTT, to distinguish prey-infested plants from non-prey infested plants.

## Conclusion

Application of a low dose of the phytohormone JA results in augmented transcript levels of a terpene biosynthetic gene and emission of a volatile metabolite crucial in plant indirect defence, when followed by a minor infestation of herbivores. This synergistic effect is observed irrespective of whether phytohormone and infestation occur simultaneously or sequentially, and might lead to a memory effect of plant indirect defence. Phytohormone application has thus the potential to induce enhanced biological pest control against spider mites. Moreover, this study provides information that indirect defence is stable in case of simultaneous and sequential attack by herbivores that induce similar signal transduction pathways in plants and may even be enhanced in the presence of multiple herbivores. However, the effect on other tritrophic interactions, other plants species, and the persistence of this effect require further investigation.

## Supplementary data

Supplementary data are available at *JXB* online.

Figure S1. Relative gene transcript levels of *PlOS* of 3 independent experiments spaced in time, quantified in *P. lunatus* plants treated with (i) water (control), (ii) 0.1mM JA, (iii) four *T. urticae* (water+4Tu), or (iv) 0.1mM JA with four *T. urticae* mites (0.1mM JA + 4Tu). Simultaneous application of four *T. urticae* on plants for 48h. Values are the mean (± SE) of ten to twelve biological replicates, different letters above bars indicate significant differences in transcript levels between treatments (Fisher’s LSD tests, α=0.05). *PlOS* transcript levels were normalized to the normalization factor obtained from geometrically averaging the Ct values of the two reference genes *PlACT1* and *PlNMP1* for each sample. Baseline represents transcript level in control plants.

Figure S2. Relative gene transcript levels of *PlOS* of two experiments spaced in time, quantified in *P. lunatus* plants treated with (i) water (control), (ii) 0.1mM JA, (iii) four *T. urticae* (water+4Tu), or (iv) 0.1mM JA with four *T. urticae* mites (0.1mM JA + 4Tu). Sequential application of four *T. urticae* placed on plants for 48h after prior application with water or 0.1mM JA 7 days before. Values are the mean (± SE) of six to eight biological replicates, different letters above bars indicate significant differences in transcript levels between treatments (Fisher’s LSD tests, α=0.05). *PlOS* transcript levels were normalized to the normalization factor obtained from geometrically averaging the Ct values of the two reference genes *PlACT1 and PlNMP1* for each sample. Baseline represents transcript level in control plants.

Figure S3. SA levels in ng SA per g FW in *P. lunatus* plants treated with (i) water (control), (ii) 0.1mM JA, (iii) four *T. urticae* (water + 4Tu), or (iv) 0.1mM JA with four *T. urticae* mites (0.1mM JA+4Tu). (A) Inoculation of four adult female *T. urticae* on plants was done immediately following JA-treatment and mites had since been feeding for 48h, and (B) inoculation of four adult female *T. urticae* for 48h was done 7 days after incubation with water or 0.1mM JA started and mites had since been feeding for 48h. Values are the mean (± SE) of four biological replicates, and were analysed by ANOVA (A) or Kruskal-Wallis test (B) respectively (α = 0.05).

Supplementary Data
