# Supplementary material for: Synergism in the effect of prior jasmonic acid application on herbivore-induced volatile emission by Lima bean plants: transcription of a monoterpene synthase gene and volatile emission
Source: J Exp Bot. 2014 Jun 13;65(17):4821–31. doi: 10.1093/jxb/eru242 (PMC4144767; doi:10.1093/jxb/eru242)
Supplement: Supplementary Data [file supp_65_17_4821__index.html]

Synergism in the effect of prior jasmonic acid application on herbivore-induced volatile emission by Lima bean plants: transcription of a monoterpene synthase gene and volatile emission — Synergism in the effect of prior jasmonic acid application on herbivore-induced volatile emission by Lima bean plants: transcription of a monoterpene synthase gene and volatile emission — Supplementary Data 

# Synergism in the effect of prior jasmonic acid application on herbivore-induced volatile emission by Lima bean plants: transcription of a monoterpene synthase gene and volatile emission

## Supplementary Data

Data files

**Files in this Data Supplement:**

- Supplementary Data - Supplementary Data
