# Supplementary material for: Synergism in the effect of prior jasmonic acid application on herbivore-induced volatile emission by Lima bean plants: transcription of a monoterpene synthase gene and volatile emission
Source: J Exp Bot. 2014 Jun 13;65(17):4821–31. doi: 10.1093/jxb/eru242 (PMC4144767; doi:10.1093/jxb/eru242)
Supplement: Supplementary Data [file supp_eru242_jexbot121301_file001.pdf]

Synergism in the effect of prior jasmonic acid application on herbivore-induced volatile emission by Lima bean plants: transcription of a monoterpene synthase gene and volatile emission

Tila R. Menzel[1], Berhane T. Weldegergis[1], Anja David[2], Wilhelm Boland[2], Rieta Gols[1], Joop J.A. van Loon[1], Marcel Dicke[1]*

1) Laboratory of Entomology, Wageningen University, P.O. Box 8031, 6700 EH, Wageningen, The Netherlands
2) Max Planck Institute for Chemical Ecology, Hans Knoell Strasse 8, D-07745 Jena, Germany

*To whom correspondence should be addressed. E-mail: marcel.dicke@wur.nl

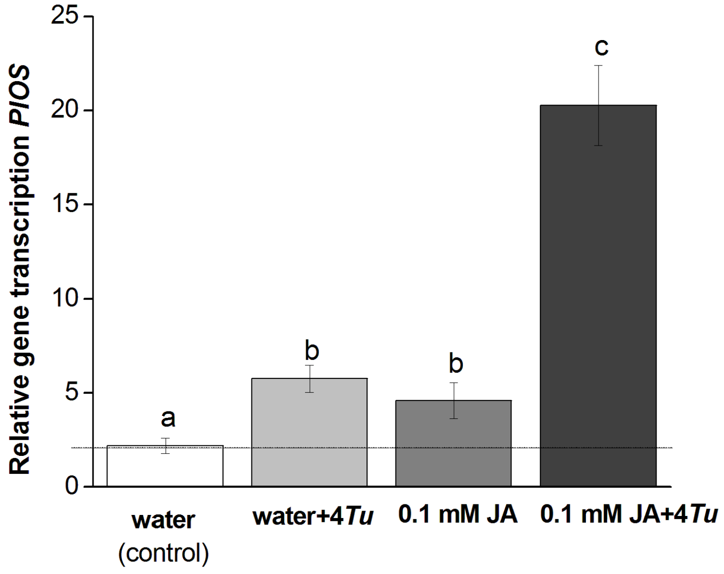

Supplemental Figure 1. Relative gene transcript levels of *PlOS* of 3 independent experiments spaced in time, quantified in *P. lunatus* plants treated with i) water (control), ii) 0.1 mM JA, iii) four *T. urticae* (water + 4*Tu*), or iv) 0.1 mM JA with four *T. urticae* mites (0.1 mM JA + 4*Tu*). Simultaneous application of four *T. urticae* on plants for 48 h. Values are the mean (± SE) of ten to twelve biological replicates, different letters above bars indicate significant differences in transcript levels between treatments (Fisher's LSD tests, α = 0.05). *PlOS* transcript levels were normalized to the normalization factor obtained from geometrically averaging the Ct values of the two reference genes *PlACT1 and PlNMP1* for each sample. Baseline represents transcript level in control plants.

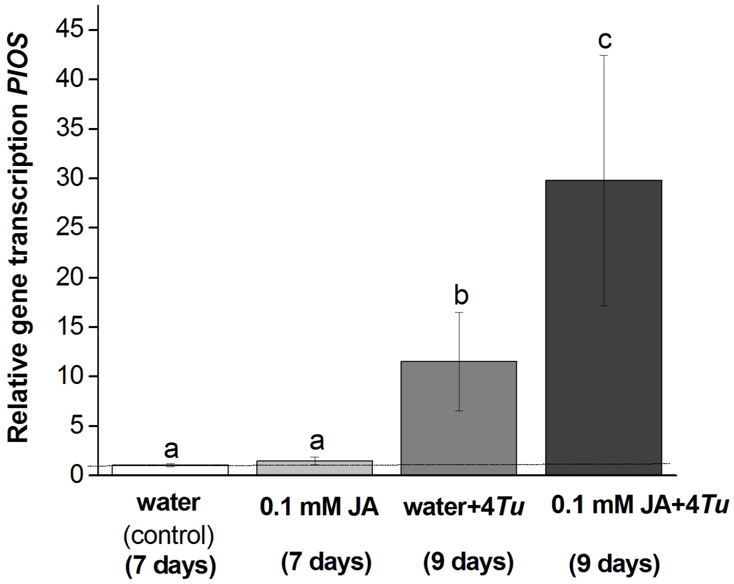

Supplemental Figure 2. Relative gene transcript levels of *PlOS* of 2 experiments spaced in time, quantified in *P. lunatus* plants treated with i) water (control), ii) 0.1 mM JA, iii) four *T. urticae* (water + 4*Tu*), or iv) 0.1 mM JA with four *T. urticae* mites (0.1 mM JA + 4*Tu*). Sequential application of four *T. urticae* placed on plants for 48 h after prior application with water or 0.1 mM JA seven days before. Values are the mean (± SE) of six to eight biological replicates, different letters above bars indicate significant differences in transcript levels between treatments (Fisher's LSD tests, α = 0.05). *PlOS* transcript levels were normalized to the normalization factor obtained from geometrically averaging the Ct values of the two reference genes *PlACT1 and PlNMP1* for each sample. Baseline represents transcript level in control plants.

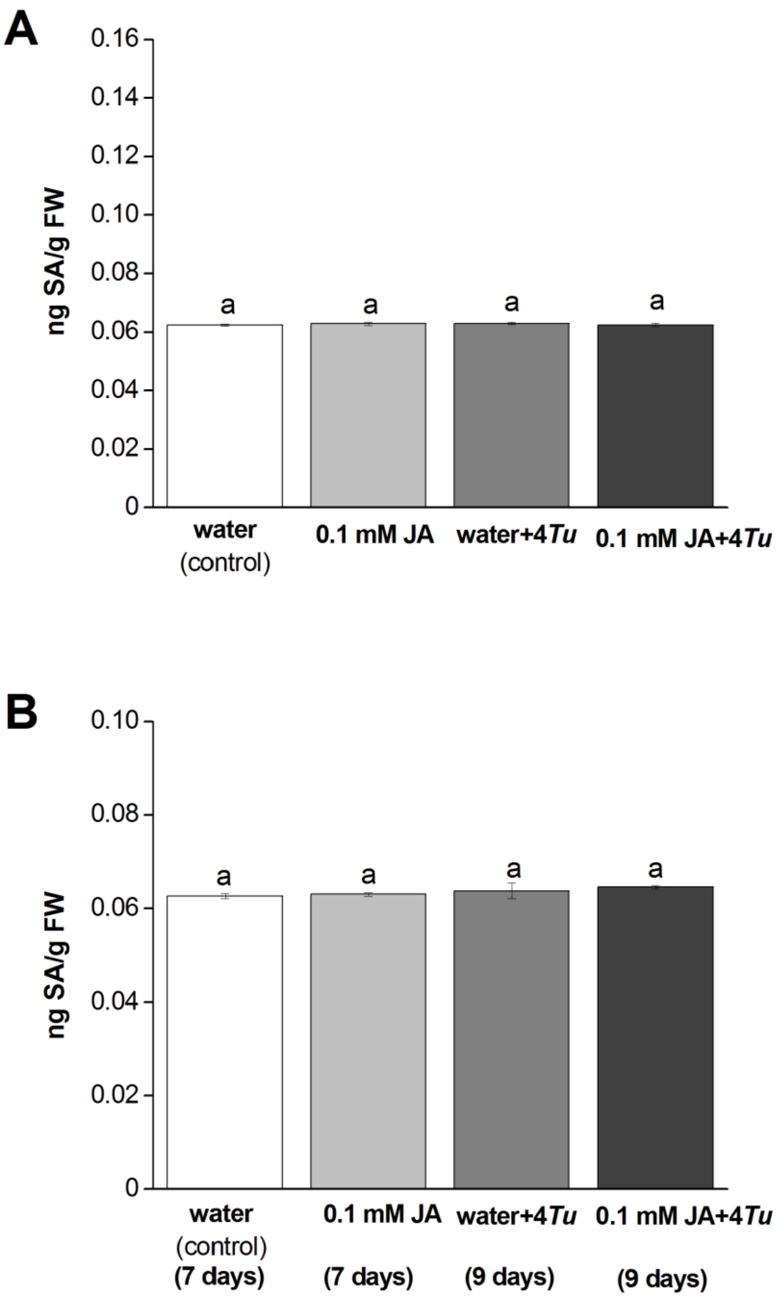

Supplemental Figure 3. SA levels in ng SA/g FW in *P. lunatus* plants treated with i) water (control), ii) 0.1 mM JA, iii) four *T. urticae* (water + 4*Tu*), or iv) 0.1 mM JA with four *T. urticae* mites (0.1 mM JA + 4*Tu*). (A) Inoculation of four adult female *T. urticae* on plants was done immediately following JA-treatment and mites had since been feeding for 48 h, and (B) inoculation of four adult female *T. urticae* for 48 h was done seven days after incubation with water or 0.1 mM JA started and mites had since been feeding for 48 h. Values are the mean (± SE) of four biological replicates, and were analysed by ANOVA (A) or Kruskal-Wallis test (B) respectively (α = 0.05).
